# Development of a recombinase polymerase amplification assay for rapid detection of *Haemophilus parasuis* in tissue samples

**DOI:** 10.1002/vms3.287

**Published:** 2020-05-26

**Authors:** Qiaoyi Han, Jinfeng Wang, Ruiwen Li, Qingan Han, Wanzhe Yuan, Jianchang Wang

**Affiliations:** ^1^ College of Veterinary Medicine Hebei Agricultural University Baoding China; ^2^ Technology Center of Shijiazhuang Customs Shijiazhuang China; ^3^ Hebei Academy of Science and Technology for Inspection and Quarantine Shijiazhuang China; ^4^ Hebei Animal Disease Prevention and Control Center Shijiazhuang China

**Keywords:** diagnosis, Glässer's disease, *Haemophilus parasuis*, *infB* gene, real‐time RPA

## Abstract

*Haemophilus parasuis* is the etiological agent of Glässer's disease in swine, which associates with severe economic losses in the swine industry worldwide. A real‐time recombinase polymerase amplification assay (real‐time RPA) was developed for direct and rapid detection of *H. parasuis* basing on the translation‐initiation factor IF2 (*infB*) gene. The assay was performed successfully at 39°C for 20 min in Genie III, which is portable and chargeable by battery. The developed assay was highly specific for *H. parasuis*, and the limit of detection of the assay was 6.0 × 10^3^ fg of *H. parasuis* genomic DNA, which was the same as that of a real‐time PCR developed previously. The assay was further evaluated on 68 pig tissue samples, and 18 (26.5%), 20 (29.4%), and 8 (11.8%) samples were positive for *H. parasuis* by the real‐time RPA, real‐time PCR and bacterial isolation, respectively. With the bacteria isolation as the reference method, the real‐time RPA showed a diagnostic specificity of 83.33% and a diagnostic sensitivity of 100%. The above data demonstrated the well‐potentiality and usefulness of the developed real‐time RPA assay in reliable diagnosis of swine Glässer's disease, especially in resource limited settings.

## Graphical abstract

*Haemophilus parasuis* is the etiological agent of Glässer’s disease in swine, which associates with severe economic losses in the swine industry worldwide. A real‐time recombinase polymerase amplification assay (real‐time RPA) was developed for direct and rapid detection of *H. parasuis* basing on the infB gene.

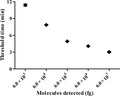

## 1 INTRODUCTION

*Haemophilus parasuis*, recently renamed *Glaesserella parasuis* after detailed phylogenetic analysis, is a non‐motile, pleomorphic, gram‐negative bacillus of the Pasturellacea family, and is the nicotinamide adenine dinucleotide (NAD) dependent bacterium (Dickerman, Bandara, & Inzana, 2020; Zhang, Tang, Liao, & Yue, 2014). *Haemophilus parasuis* is the etiological agent of Glässer's disease, which is characterized by fibrinous polyserositis, peritonitis, polyarthritis and meningitis (Oliveira & Pijoan, 2004). Presently, 15 serovars of *H. parasuis* have been identified worldwide (McCaig, Loving, Hughes, & Brockmeier, 2016), and the most common serovars in China are serovars 4 and 5 (Cai et al., 2005; Ma et al., 2016; Zhang, Xu, et al., 2012). *Haemophilus parasuis* could cause a infection rate of 50%–70% and a mortality rate above 10%, thus inducing considerable production losses due to the mortality and unthrifty of pigs (McCaig et al., 2016; Zhang et al., 2014). Glässer's disease has caused severe economic losses in the swine industry worldwide.

The *H. parasuis* infection can be controlled by vaccination and antibiotic treatment. However, one of the key elements for controlling the *H. parasuis* infection is the rapid and accurate detection of the bacterium (Aarestrup, Seyfarth, & Angen, 2004; Oliveira & Pijoan, 2004). Isolation and microbiological culture of *H. parasuis* is the gold standard for diagnosis of Glässer's disease, but it could be ineffective due to the fastidious growth of the bacteria, easily being overgrown by other bacterial contaminants and the previous antibiotic treatment of affected animals (Angen, Oliveira, Ahrens, Svensmark, & Leser, 2007, Oliveira et al., 2001, Turni, Pyke, & Blackall, 2010). A series of molecular diagnostic methods have been described for sensitive and specific detection of *H. parasuis*, such as polymerase chain reaction (PCR), real‐time PCR, loop‐mediated isothermal amplification (LAMP) and cross‐priming amplification (CPA) (Angen et al., 2007, Chen, Chu, Liu, Zhang, & Lu, 2010, Frandoloso, Martinez‐Martinez, Rodriguez‐Ferri, & Gutierrez‐Martin, 2012, Gou et al., 2018, Oliveira et al., 2001, Turni et al., 2010, Yang et al., 2010). The requirements of high‐precision thermocycler, a centralized laboratory facility and experienced technicians limit the widespread use of PCR assays in under‐equipped laboratories, in clinic settings, and on farm diagnosis. Although the developed LAMP assay shows the advantages in respects to convenience and minimal equipment requirement, it still requires four primers, 60 min at 61°C or 45 min at 65°C for reaction and 10 min at 80°C to terminate the reaction (Chen et al., 2010; Yang et al., 2010). The CPA assay combines with a vertical flow visualization nucleic acid detection strip showing good specificity and sensitivity, thus reducing the requirement of specialized instrument, however, the reaction time is 1h (Gou et al., 2018). A rapid test may assist the microbiological diagnosis for peritonitis in pigs, so a more convenient and rapid detection method is still required for detection of *H. parasuis* on farm and in low resource settings.

Recombinase polymerase amplification (RPA), which is an isothermal DNA amplification technique, has experienced rapid development in the field of molecular diagnostic since it was firstly reported in 2006 (Daher, Stewart, Boissinot, & Bergeron, 2016; Lei et al., 2020; Li, Macdonald, & von Stetten, 2018; Liu et al., 2018; Miao et al., 2019; Piepenburg, Williams, Stemple, & Armes, 2006; Wang, Yuan, Han, Wang, & Liu, 2017). Some studies showed that RPA may be the most applicable approach for the on farm diagnosis due to its convenience and rapidness (Amer et al., 2013; Li et al., 2018). In this study, the real‐time RPA assay targeting the translation‐initiation factor IF2 (*infB*) gene was developed for rapid and reliable detection of *H. parasuis*, and the performance of the assay was further evaluated and compared to the bacteria isolation and a real‐time PCR by detecting the swine tissue samples.

## 2 MATERIALS AND METHODS

### 2.1 Bacteria strains and clinical samples

To determine the specificity of the real‐time RPA, a total of 20 *H. parasuis* strains and 17 other bacterial strains were tested. For the *H. parasuis* strains, the genomic DNA of 15 serovar reference strains were kindly provided by Lanzhou Veterinary Research Institute (Lanzhou, China), two reference strains were obtained from CIVDC (China Institute of Veterinary Drug Control, Beijing, China), and three field strains were identified by species‐specific PCR described previously (Oliveira et al., 2001, Turni et al., 2010). Details of all the bacterial strains used in this study were provided in Table 1.

**Table 1:** Bacterial strains used in this study

Organism	Serovar	Reference/field strain	Detection results	
			Real‐time RPA	Real‐time PCR
*Haemophilus parasuis*	1	NO.4	+	+
	2	SW140	+	+
	3	SW114	+	+
	4	CVCC3894	+	+
		SW124	+	+
	5	CVCC3895	+	+
		Nagasaki	+	+
	6	131	+	+
	7	174	+	+
	8	C5	+	+
	9	D74	+	+
	10	H367	+	+
	11	H465	+	+
	12	H425	+	+
	13	17,975	+	+
	14	22,113	+	+
	15	15,995	+	+
	ND	Field strain	+	+
	ND	Field strain	+	+
	ND	Field strain	+	+
*Actinobacillus pleuropneumoniae*	1	CVCC259	−	−
	3	CVCC261	−	−
	5	CVCC263	−	−
	7	CVCC265	−	−
	8	CVCC266	−	−
*Pseudomonas aeruginosa*		CICC21636	−	−
		Field strain	−	−
*Streptococcus hemolytic‐β*		CMCC10373	−	−
*Bacillus cereus*		ATCC 14579	−	−
		Field strain	−	−
*Pasteurella multocida*		Field strain	−	−
		Field strain	−	−
*Bordetella bronchiseptica*		Field strain	−	−
*Mycoplasma hyopneumoniae*		Strain 168	−	−
*Klebsiella pneumoniae*		ATCC 4352	‐	‐
		Field strain	‐	‐
*Mannheimia haemolytwa*		Field strain	‐	‐
*Listeria monocytogenes*		ATCC 15313	−	−
		Field strain	−	−
*Staphylococcus aureus*		ATCC 6538	−	−
		Field strain	−	−

Abbreviations: −, negative; +, positive; ATCC, America Type Culture Collection; CICC, China Center of Industrial Culture Collection; CMCC, National Center for Medical Culture Collection; CVCC, China Veterinary Culture Collection Center; ND, not determined.

Thirty swine tonsils were collected at slaughterhouse, and 38 swine fresh lungs were collected from the markets for agricultural products in Shijiazhuang city, Hebei province from November to December 2018.

### 2.2 DNA extraction

All the bacterial genomic DNA were extracted using the TIANamp Bacteria DNA kit (Tiangen), which were performed according to the manufacturer's instructions. The tonsil and lung samples were homogenized with phosphate‐buffered saline (PBS, pH 7.4) as a 10% (w/v) suspension and centrifuged for 10 min at 10,000 g at 4°C. After centrifugation, the supernatant was discarded and the remaining pellet was suspended in 200 μl of PBS for DNA extraction using the TIANamp Bacteria DNA kit (Tiangen). Total DNA extracted from tissue samples was finally eluted in 50 µl of nuclease‐free water. All DNA were quantified using a ND‐2000c spectrophotometer (NanoDrop) and stored at −80°C until use.

### 2.3 Real‐time RPA primers and probes

The *infB* gene is highly conserved in all serovars of *H. parasuis* and is determined as the detection target of real‐time RPA. Nucleotide sequence data for all 15 serovars of *H. parasuis* strains available in GenBank were aligned to identify the conserved regions in the *infB* gene according to the reference sequences of *H. parasuis* (accession number: HM243087, DQ781806, DQ781808, DQ781810–DQ781817, CP001321.1, CP005384.1), the primers and exo probe were designed following the RPA manufacturer guidelines (TwistDx), and the amplicon size is 182 bp. Primers and probe are listed in Table 2 and synthesized by Sangon Biotech Co., Shanghai, China.

**Table 2:** Sequences of the primers and probes for *Haemophilus parasuis* real‐time RPA and PCR assays

Assay	Primers and probes	Sequence 5´−3´	Amplicon size (bp)	References
Real‐time RPA	infB‐exo‐F	ACCAGAAGCAAACCTAGAGCGTGTAGAGCAA	182	This study
	infB‐exo‐R	CCTCTTTCACTGCGCTTAATTCTAATACTTCC		
	infB‐exo‐P	CACGAAGTGATTTCTGAGAAATTCGGTGG (FAM‐dT)G(THF)(BHQ1‐dT)GTTCAATTTGTTCC ‐C3spacer		
Real‐time PCR	CTinfF1	CGACTTACTTGAAGCCATTCTTCTT	75	Turni et al. (2010)
	CTinfR1	CCGCTTGCCATACCCTCTT		
	CTinfP	FAM‐ ATCGGAAGTATTAGAATTAAGTGC ‐TAMRA		
PCR	HPS‐forward	GTGATGAGGAAGGGTGGTGT	821	Oliveira et al. (2001)
	HPS‐reverse	GGCTTCGTCACCCTCTGT		

### 2.4 Real‐time RPA assay

The *H. parasuis* RPA assay was performed using a ZC BioScience^TM^ exo kit (ZC BioScience, Hangzhou, China). Firstly, the freeze‐dried enzyme pellet in the reaction tube was rehydrated by 40.5 μl of rehydration buffer. Then, other reaction components including 2.1 μl of forward primer (infB‐exo‐F, 10 μM), 2.1 μl of reverse primer (infB‐exo‐R, 10 μM), 0.8 μl of exo probe (ecfX‐exo‐P, 10 μM), 1 μl of genomic DNA and 1 μl of ddH2O were added into the reaction tube and vortexed thoroughly. Finally, 2.5 μl of magnesium acetate (280 mM) was added into the reaction tube and the total reaction volume was 50 μl. The reaction tube was vortexed briefly and spun down, and the assay was performed immediately at 39°C for 20 min in a Genie III scanner device (OptiGene Limited). Samples produced an exponential amplification curve above the threshold of the negative control were considered positive.

### 2.5 Analytical specificity and sensitivity of the real‐time RPA assay

The analytical specificity of *H. parasuis* real‐time RPA assay was determined by testing the genomic DNA extracted from a panel of bacteria listed in Table 1. Three independent reactions were performed.

The analytical sensitivity of *H. parasuis* real‐time RPA assay was determined and compared with that of real‐time PCR using a 10‐fold serial dilution of genomic DNA of *H. parasuis* ranging from 6.0 × 10^7^ to 6.0 × 10^0^ fg/μl. One microliter of each dilution was amplified by RPA to determine the limit of detection (LOD) of the assay, and eight independent reactions were performed.

### 2.6 Real‐time PCR

A real‐time PCR assay for *H. parasuis* was performed on a ABI 7500 instrument described previously (Turni et al., 2010). Sequences for the primers (CTinfF1 and CTinfR1) and TaqMan probe (CTinfP) are provided in Table 2. The Premix Ex Taq (Takara) was applied in real‐time PCR assay and the reaction was performed as follows: 95°C for 30 s; then 40 cycles of 95°C for 10 s and 60°C for 40 s.

### 2.7 Validation with tissue samples

The *H. parasuis* real‐time RPA assay were assessed on 38 swine fresh lungs and 30 swine tonsils, and all samples tested by RPA assay were also tested by a real‐time RT‐PCR (Turni et al., 2010), which was run in parallel. Bacteria isolation of the *H. parasuis* was also performed for the above tissue samples, which was performed in detail as the following protocol. The surfaces of the tonsils and lungs were seared with a hot iron and cut to obtain scrapings of sample tissue for culture. Scraping of tissue was spread on TSA plates supplemented with 5% foetal bovine serum and 0.01% NAD (Sigma). The plates were incubated at 37°C for 48 hr. All suspect colonies of *H. parasuis* were passaged twice before identification by PCR (Oliveira et al., 2001).

## 3 RESULTS

### 3.1 Analytical specificity of the real‐time RPA assay

Specific amplification was only observed with *H. parasuis*, including the reference strains and the field isolates, and there was no cross‐detections of other bacteria tested (Table 1). Three independent reactions were repeated and similar results were observed, which demonstrated the high specificity and good repeatability of the assay.

### 3.2 Analytical sensitivity of the real‐time RPA assay

**Figure 1:**
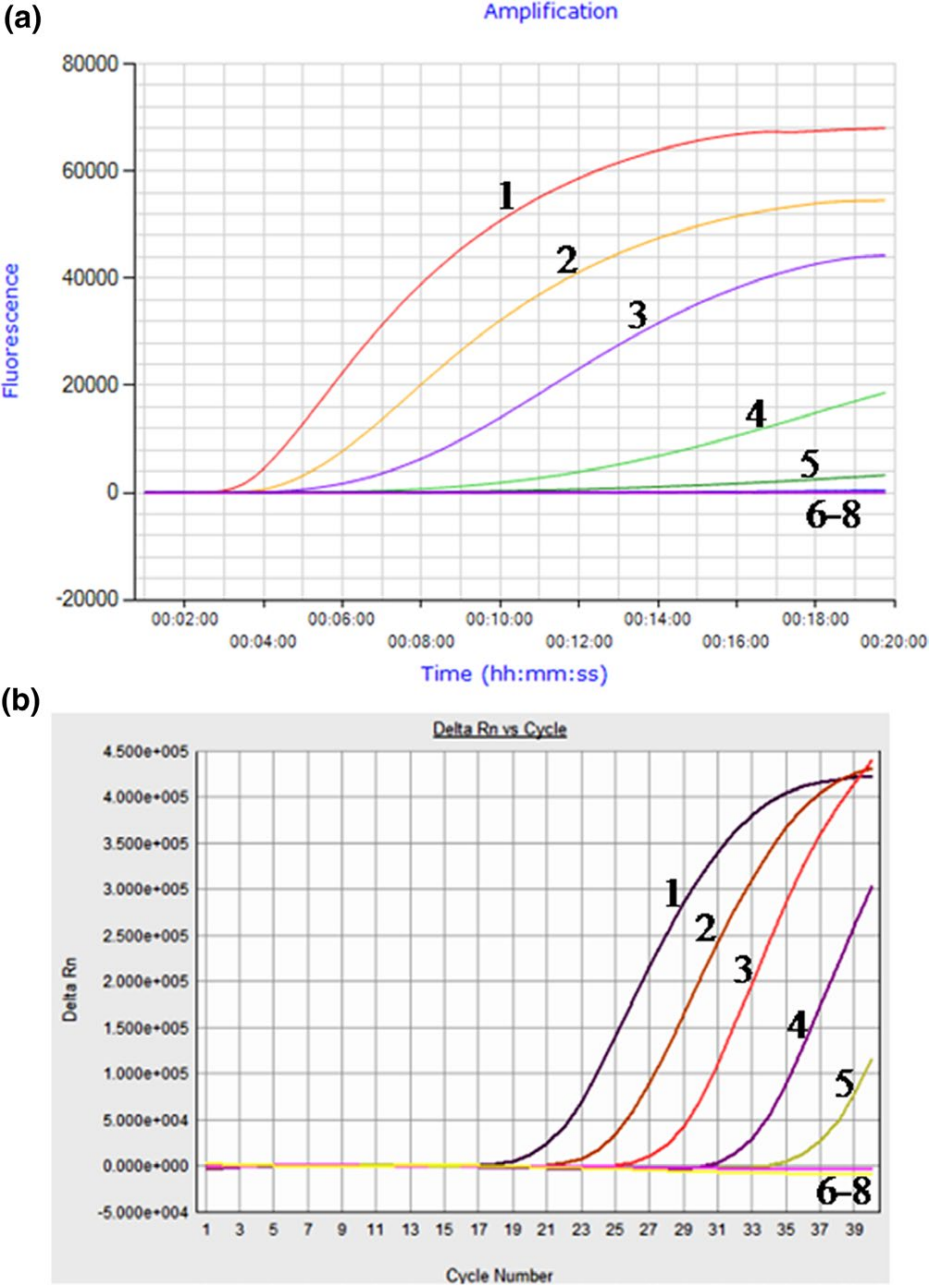
Comparative analytical sensitivity of real‐time RPA and real‐time PCR assays for *Haemophilus parasuis*. The assays were performed using the 10‐fold dilution of *H. parasuis* genomic DNA from 6.0 × 10^7^fg to 6.0 × 10^0^ fg per tube, and the assays showed the same analytical sensitivity, 6.0 × 10^3^ fg. A: Limit of detection of real‐time RPA assay with a Genie III. B: Limit of detection of real‐time PCR assay with an ABI 7500 system. Lane 1. 6.0 × 10^7^ fg; lane 2. 6.0 × 10^6^ fg; lane 3. 6.0 × 10^5^ fg; lane 4. 6.0 × 10^4^ fg; lane 5. 6.0 × 10^3^ fg; lane 6. 6.0 × 10^2^ fg; lane 7. 6.0 × 10^1^ fg; lane 8. 6.0 × 10^0^ fg

**Figure 2:**
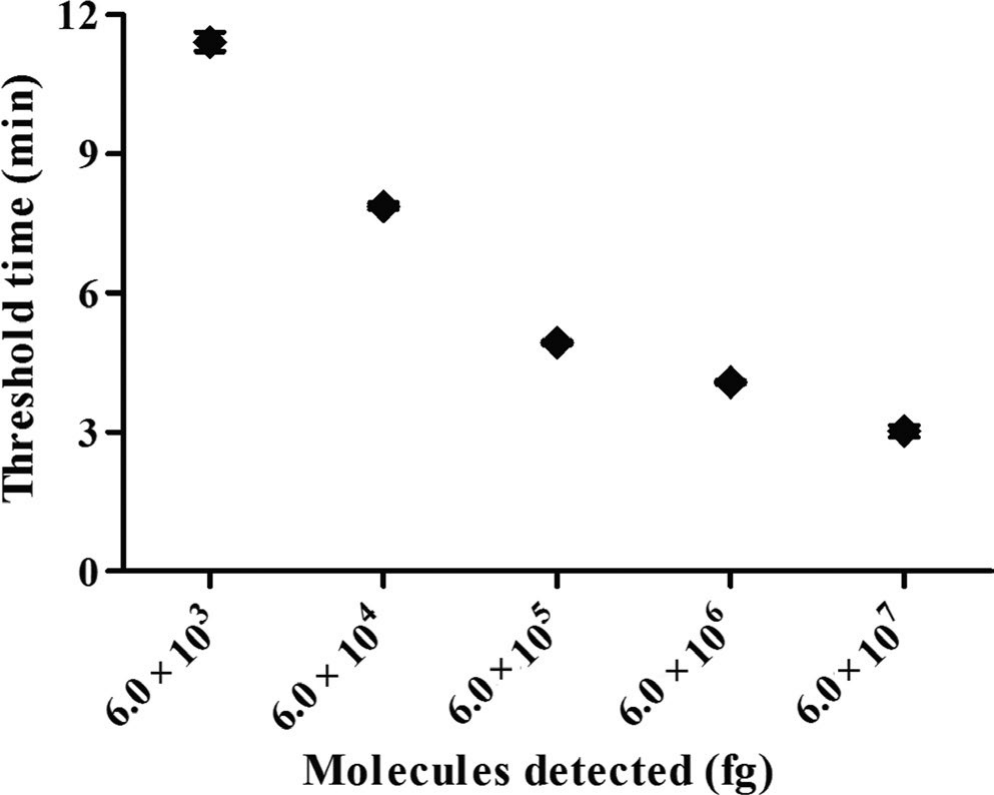
Reproducibility of *Haemophilus parasuis* real‐time RPA assay. Semi‐logarithmic regression of the data collected from eight real‐time RPA runs tested on the *H. parasuis* genomic DNA standards using Prism Software. The run time of the assay was between 3 and 12 min for 6.0 × 10^7^ fg‐6.0 × 10^3^ fg genomic DNA

Using a dilution range of 6.0 × 10^7^ to 6.0 × 10^0^ fg of *H. parasuis* genomic DNA as template, the data showed that the LOD of the real‐time RPA assay was 6.0 × 10^3^ fg, which was the same as that of the real‐time PCR (Figure 1). The real‐time RPA assay was performed eight times on the molecular standard, in which 6.0 × 10^7^–6.0 × 10^3^ fg DNA molecules were detected in 8/8 runs, and 6.0 × 10^2^–6.0 × 10^0^, 0/8 (Figure 2). Two‐fold serial dilutions of the genomic DNA were made from 6.0 × 10^3^ to 7.5 × 10^2^ fg/μl. The above twofold dilutions of the genomic DNA were further used in the RPA, and the LOD of the assay was still 6.0 × 10^3^ fg per reaction.

### 3.3 Validation of the real‐time RPA assays on tissue samples

Of the 68 swine tissue samples, 18 (26.5%), 20 (29.4%) and 8 (11.8%) samples were positive for *H. parasuis* by the real‐time RPA, real‐time PCR and bacterial isolation, respectively (Table 3). All the positive samples by bacterial isolation were also positive by both real‐time RPA and real‐time PCR. The Ct values of two samples tested positive in real‐time PCR while negative in real‐time RPA were over 35.0, which contained low amounts of *H. parasuis* DNA. Compared to the bacteria isolation, the real‐time RPA assay showed a diagnostic sensitivity (DSe) of 100%, a diagnostic specificity (DSp) of 83.33%, a positive predictive value (PPV) of 44.44% and a negative predictive value (NPV) of 100% (Table 4). It took approximately 5–15 min in the real‐time RPA assay to obtain the positive results, while it took approximately 30–50 min in the real‐time PCR with the Ct values ranging from 22.73 to 36.65. The above data indicated that the performance of the real‐time RPA assay was comparable to the real‐time PCR, but the real‐time RPA assay is faster.

**Table 3:** Comparison of *Haemophilus parasuis* bacteriology, real‐time RPA and real‐time PCR assays for detection of tissue samples

Samples	Number of samples	Real‐time RPA		Real‐time PCR		Bacteriology	
		P	N	P	N	P	N
Tonsil	30	5	25	7	23	2	28
Lung	38	13	25	13	25	6	32
T	68	18	50	20	48	8	60

Abbreviations: P, positive; N, negative; T, total.

**Table 4:** Diagnostic sensitivity, diagnostic specificity and predictive values of real‐time RPA assay and bacteria isolation method for detection of *Haemophilus parasuis*

	Bacteria isolation		
	P	N	T
Real‐time RPA			
P	8	10	18
N	0	50	50
T	8	60	68
	DSe: 100%	DSp: 83.33%	
	PPV: 44.44%	NPV: 100%	

Abbreviations: DSe, diagnostic sensitivity; DSp: diagnostic specificity; N, negative; NPV, negative predictive value.; P, positive; PPV, positive predictive value; T, total.

## 4 DISCUSSION

Although isolation of *H. parasuis* is the gold standard for diagnosis of Glässer's disease, but it is usually difficult as the bacteria is very sensitive to pH changes and heat (Morozumi & Hiramune, 1982) and it is also a slow growing, fastidious organism with specific nutritional requirements (Angen et al., 2007, Oliveira et al., 2001, Turni et al., 2010). Furthermore, *H. parasuis* is easily overgrown by other faster growing bacteria. This makes recovery of the *H. parasuis* very difficult after sample collection and transport to the laboratory. Therefore, the method of identification by culture is not always optimal, and nucleic acid amplification‐based methods are the attractive alternatives. In this study, we developed a real‐time RPA assay for rapid detection of *H. parasuis*, and the positive results could be obtained within 5–15 min. The assay for *H. parasuis* was performed on the Genie III, which is portable, lightweight and could work a whole day charging by battery. The assay was further demonstrated to be specific, sensitive and easy to perform in the detection of the swine lung and tonsil samples. RPA reagents are provided in the form of lyophilized powder and cold chain independent, and RPA is tolerant to most of the PCR inhibitors during the amplification process (Daher et al., 2016; Lillis et al., 2016; Moore & Jaykus, 2017). The above facts make the real‐time RPA assay a good potentiality of rapid detection of *H. parasuis* in the farm conditions, which could clarify the microbiology in a difficult diagnostic situation of serositis in pigs.

Several studies had demonstrated the efficacy of the PCR and LAMP to detect *16S rRNA, infB* gene and other conserved regions of genomic DNA of *H. parasuis* in different clinical specimens (Angen et al., 2007, Chen et al., 2010, Gou et al., 2018, McDowall, Slavic, MacInnes, & Cai, 2014, Oliveira et al., 2001, Turni et al., 2010, Yang et al., 2010). The PCR targeting on *16S rRNA* gene has problems in specificity giving a weak positive with *Actinobacillus indolicus* (Turni et al., 2010), and a real‐time PCR using the *16S rRNA* as the target could not differentiate *Pasteurella mairii* from *H. parasuis* (Turni et al., 2010). The *infB* gene codes for the two forms of the translation initiation factor IF2 – IF2 alpha and IF2 beta (Hedegaard et al., 2000). The developed real‐time PCR and LAMP based on the *infB* gene could detect all the 15 serovars of *H. parasuis* and demonstrated good performance (Chen et al., 2010; Turni et al., 2010; Zhang, Shen, et al., 2012), which shows that the *infB* gene is a good target for molecular detection methods for *H. parasuis* and could separate *H. parasuis* from all other closely related species. In this study, the real‐time RPA primers and probe were also designed based on the *infB* gene in this study, just as the PCR and LAMP assays developed previously (Chen et al., 2010; Turni et al., 2010; Zhang, Shen, et al., 2012), and the real‐time RPA assay demonstrated very good specificity in the detection of *H. parasuis*.

Of the 68 tissue samples, 18 (26.5%) and 20 (29.4%) samples were positive for *H. parasuis* by the real‐time RPA and real‐time PCR, respectively, which were much higher than the bacterial isolation (8, 11.8%). Compared to the bacterial isolation, the PPV and NPV of real‐time RPA was 44.44% and 100%. All the negative samples in real‐time RPA were also negative in bacteria isolation, while the *H. parasuis* could be only isolated in less than half of the positive samples in real‐time PRA. One possible reason is that the bacteria isolation of *H. parasuis* is difficult, especially in the tissue samples collected in the slaughterhouse and market. It is also possible that only the dead *H. parasuis* or genomic DNA but not the live bacteria were present in the samples. The low positive predictive value of RPA was a deficiency of this study, and the assay should be assessed on more clinical positive samples in the following study. The diagnostic performance of the real‐time RPA assay for *H. parasuis* was comparable to the real‐time PCR assay, while the RPA shows distinct advantages of rapidness and convenience. Therefore, we believe that real‐time RPA will enhance the diagnosis of *H. parasuis* infection, especially for those laboratories without access to real‐time PCR instrumentation, those are not experienced in the culture of *H. parasuis* or in situation where culture is not possible.

In summary, the developed real‐time RPA assay is rapid and reliable for detection of *H. parasuis*, and demonstrates great promise in the diagnosis of Glässer's disease in laboratory and in the field, which is of great importance to eliminate the infected pigs from the herds.

## CONFLICT OF INTEREST

The authors declare that they have no competing interests.

## AUTHOR CONTRIBUTION

Qiaoyi Han: Data curation; Methodology. Jinfeng Wang: Methodology. Ruiwen Li: Data curation; Validation. Qingan Han: Funding acquisition; Resources. Wanzhe Yuan: Conceptualization; Funding acquisition; Writing‐review & editing. Wang Jianchang: Conceptualization; Funding acquisition; Supervision; Writing‐original draft.

## ETHICAL STATEMENT

The authors confirm that the ethical policies of the journal, as noted on the journal's author guidelines page, have been adhered to and the appropriate ethical review committee approval has been received. The US National Research Council's guidelines for the Care and Use of Laboratory Animals were followed.

## Data Availability

The dataset analysed during this study is available from the corresponding author on reasonable request.
